# Frequency of* Trypanosoma cruzi* Infection in Synanthropic and Wild Rodents Captured in a Rural Community in Southeast of Mexico

**DOI:** 10.1155/2018/8059613

**Published:** 2018-10-16

**Authors:** Ivonne Hernández-Cortazar, Karla Cecilia Amaya Guardia, Marco Torres-Castro, Karla Acosta-Viana, Eugenia Guzmán-Marín, José Israel Chan-Pérez, Antonio Ortega-Pacheco, Roger I. Rodríguez-Vivas, Rodrigo Medina-Pinto, Matilde Jiménez-Coello

**Affiliations:** ^1^Laboratorio de Biología Celular, Centro de Investigaciones Regionales “Dr. Hideyo Noguchi”, Universidad Autónoma de Yucatán, Mexico; ^2^Laboratorio de Enfermedades Emergentes y Reemergentes, Centro de Investigaciones Regionales “Dr. Hideyo Noguchi”, Universidad Autónoma de Yucatán, Mexico; ^3^Campus de Ciencias Biológicas y Agropecuarias, Facultad de Medicina Veterinaria y Zootecnia, Universidad Autónoma de Yucatán, Mexico

## Abstract

The protozoan parasite* Trypanosoma cruzi* is the causative agent of the Chagas disease, which is endemic in southeastern Mexico and is transmitted by the vector* Triatoma dimidiata *(triatomide).* T. cruzi *infect a great variety of domestic and wild mammals; rodents are considered one of the most important reservoirs of the parasite in the transmission cycles of* T. cruzi*. The objective of this study was to determine the frequency of* T. cruzi* infection and to determine the parasitic load in synanthropic and wild rodents from the rural community of southern Mexico. A total of 41 blood samples and 68 heart tissue samples were collected from various species of synanthropic (n= 48 in 2 species) and wild rodents (n= 35 in 5 species). DNA was extracted from samples to detect the presence of* T. cruzi *through quantitative PCR (qPCR).* T. cruzi *DNA was detected in the 9.75% of the blood samples of the synanthropic species (4/41) (14.28%) for* Rattus rattus* samples and 25% for* Ototylomys phyllotis* samples, with an average of parasitic load of 4.80 ± 1.17 parasites/*μ*L. In the case of heart tissue samples, 10.29% were positive for* T. cruzi* (7/68) (8.7% for* Rattus rattus*, 40% for* Peromyscus yucatanicus,* and 42.8% for* Ototylomys phyllotis*) with an average parasite load of 3.15 ± 1.98 eq-parasites/mg. The active and chronic infection of* T. cruzi* in synanthropic or wild rodents of the rural community of southern Mexico evidences the natural infection in these reservoirs which contribute to maintaining the agent in the wild and domestic environments and can represent a risk of infection for the human population when the vector is present.

## 1. Introduction

Rodents are the most abundant and diverse group of mammals, accounting for 43% of all mammalian species. In many regions of the world, rodents live in close contact with human populations because human activity creates suitable habitats for the prosperity of these animals (availability of food and shelter) [1]. However, human-rodent contact represents a potential risk factor for public health. It is known that they are the main reservoirs for pathogens and that they play an important role in the emergence or reemergence of different zoonotic diseases. It has been estimated that 70% of zoonoses have an origin in wildlife [2]. Although domestic and selvatic animals can be considered potential reservoirs for zoonoses, selvatic and synanthropic rodents are important because they can serve as amplifiers of pathogens and favor the dispersion of these agents in different zones [3].

Among the parasitic zoonoses with synanthropic cycles is Chagas disease, also called the American trypanosomiasis, and it is caused by the protozoan* Trypanosoma cruzi* (*T. cruzi*). This disease is distributed mainly in 21 Latin American countries and it is estimated that it affects between 7 and 8 million people. This parasite is mainly transmitted by reduviidae insects (hematophagous bugs) [4]. Due to the ability of rodents to inhabit urban or anthropized ecosystems, they represent the ideal reservoir for diseases to persist in a region. The infection of rodents with* T. cruzi* in southwestern Mexico has been documented in recent years. In this region, seropositivity has been reported in synanthropic rodents (*Rattus rattus* and* Mus musculus*) from 37.5 to 47%, suggesting that these animals act as hosts for the parasite in urban cycles [5, 6]. Although urban cycles are important for their zoonotic character, understanding the transmission dynamics of* T. cruzi* in the sylvatic and peridomestic cycles of host mammals is important for estimating the risk to humans in rural areas [7]. In addition, the identification of* T. cruzi* reservoir species that serve as sources of vector feeding could help guide strategies to reduce transmission [8].

In Mexico there is a program for the chemical control of vectors and improvement of housing to reduce infestation level inside domiciles; nevertheless, this program takes in 15 of the 32 entities of Mexico and it does not contemplate any control over the population of rodents in the urban and rural areas [9]. The rapid and disorganized growth of urban areas, which involves the invasion of the habitat of rodents and arthropod vectors and the control methods based on the vector alone, without recognizing the importance of rodents as reservoirs of the disease, makes the interaction between parasite-rodent-vector-human is increasingly close, which significantly increases the risk of infection with* T. cruzi*. Therefore, the objective of this study was to determine the presence and parasitic load of* T. cruzi* in blood and heart tissue samples from synanthropic and wild rodents captured in a rural community in southeastern Mexico.

## 2. Materials and Methods

### 2.1. Site of Study and Placement of Traps

The study was carried out in the municipality of Cenotillo, Yucatan (rural community of southeast Mexico), which is located between the parallels 20° 55′ and 21° 09′ N latitude; 88° 26′ and 88° 48′ of length W; with an altitude of 16 masl, with an average annual temperature of 26.3°C and a rainfall of 1,200 mm per year [10]. The rodents were captured in the domicile and peridomicile of the properties of the locality (synanthropic) and in small extensions of forest undisturbed by human activities located at two kilometers of the urban settlement (wild). The capture was made with the authorization of the Ministry of Environment and Natural Resources of Mexico (No. SGPA / DGVS / 00867/17) and the Bioethics Committee of the Campus of Biological and Agricultural Sciences (CCBA) of the Autonomous University of Yucatán (UADY) (No. CCBA-M-2016-004) and following the statutes of the American Society of Mammalogists (ASM) [11].

The sampling period was between July and August 2016. For the capture in the dwellings, the study location was divided into quadrants and 10 dwellings were selected for convenience (n = 40). 12 Sherman traps (8 cm x 9 cm x 23 cm; HB Sherman traps Inc; Tallahase, Florida, United States) were distributed in the interior of dwellings and outdoors (peridomicile) near evidence of activity or presence of rodent, sources of food, or accommodation. For the sampling of the forest, 100 Sherman traps were distributed along ten linear transects, placing a trap every 8 to 9 meters. The capture was made in the same days and weeks like in the urban quadrants. All the traps used were placed in the morning and checked 24 hours later. The bait used was a mixture of oat flakes and artificial vanilla flavoring.

### 2.2. Taking and Handling of Biological Samples

All captured rodents (synanthropic and wild) were moved to a suitable room for the collection of biological samples within the study site. The animals were anesthetized by an intraperitoneal injection of sodium pentobarbital (130 mg/kg) and the euthanasia was performed by cervical dislocation, according to the guidelines of the American Association of Veterinary Medicine [12] and the current Mexican regulations NOM -033-ZOO-1995. During the anesthesia, whole blood was collected via intracardiac puncture (300*μ*L) with the help of 1mL syringes (Terumo Medical Corporation®, Tokyo, Japan), the samples were deposited in microcentrifuge tubes (1.5mL) and were stored at -70°C up to the extraction of total DNA. After euthanasia, the data of the species, sex, and age of all individuals were recorded. Additionally, a necropsy was performed in order to collect heart tissue samples, which were deposited in microcentrifuge tubes (1.5*μ*L) and stored at -70°C until the total DNA extraction.

### 2.3. DNA Extraction of Blood and Heart Tissue Samples

DNA of whole blood (200 *μ*L) and heart tissue samples (25mg) were extracted using the DNeasy Blood and Tissue commercial kit (QIAGEN,2 cat # 69504), following the manufacturer's instructions.

### 2.4. Quantitative PCR (qPCR) for the Detection of* T. cruzi* Satellite DNA

A qPCR was performed to amplify a segment of 182 bp of the Sat-DNA gene of* T. cruzi*, with the sense primers TCZ-F 5′-GCTCTTGCCCACAMGGGTGC-3' and antisense TCZ-R 5′-CCAAGCAGCGGATAGTTCAGG-3′ [13]. The reaction conditions were SsoAdvanced Universal SYBR Green Supermix 1X (Bio-Rad), 0.5*μ*M of each primer and 2*μ*l of cardiac tissue DNA sample (concentration of 1 - 47ng / uL) and for the blood samples, 4 *μ*L of DNA (1-6ng) in a final volume of 20*μ*L. The amplification conditions were initial denaturation at 95°C for 15 min, followed by 50 cycles at 95°C for 10 sec, 55°C for 15 sec, and 72°C for 10 sec. The melting curve was between 74° and 95°C with increments of 0.5°C in each step. The expected melting temperature of the amplicon was 84°C. Each sample was tested in duplicate and a reaction mixture without DNA (in duplicate) was included as a negative control for each qPCR run. The parasite load was determined using standard cardiac tissue and blood curves, using serial dilutions of known amounts of* T. cruzi* epimastigotes DNA from the CL-Brener strain (1x106 to 1x10-1), in order to quantify the absolute by means of the CFX Manager Software V 2.1 (Bio-Rad). The data are presented as parasite equivalent/*μ*L blood or parasite equivalent/g tissue, respectively.

### 2.5. Data Analysis

Descriptive statistics was used to present the frequency of species captured, sex and age as well as the frequency of positivity to* T. cruzi* in blood and heart tissue samples. Additionally, 2x2 contingency tables were used to estimate the odds ratio (OR) and 95% confidence intervals (95%CI) to identify an association between the type of behavior (synanthropic or wild), sex (female or male), and age (adult or juvenile) of the rodents captured and the infection with* T. cruzi*. Analysis was made using the software Winepiscope online version [14].

## 3. Results

A total of 83 rodents were captured with the traps placed inside and outside the domiciles of the rural community of southwest Mexico. Of the total specimen's captured, 41 blood samples and 68 heart tissue samples were obtained. The frequency of capture of the synanthropic species* Rattus rattus* and* Mus musculus* was the same with 24 specimens by species, while in the case of wild rodents, five species were found, being* Heteromys gaumeri *(n = 17), the species with most capture frequency, followed by* Ototylomys phyllotis* and* Peromyscus yucatanicus* (n = 8 each one). Finally, only one rodent was captured of the species* Sigmodon toltecus* and one of the species* Peromyscus leucopus* (Table 1).

Table 2 shows the frequency of infection with* T. cruzi* diagnosed by qPCR in the samples of blood and heart tissue of synanthropic and wild rodents captured in a rural community in Yucatan, Mexico. In general,* T. cruzi* DNA was detected in the 9.75% of the blood samples (4/41) (14.28% for* Rattus rattus* samples and 25% for* Ototylomys phyllotis* samples), with an average of parasitic load of 4.80 ± 1.17 parasites/*μ*L. In the case of heart tissue samples, 10.29% were positive for* T. cruzi* (7/68) (8.7% for* Rattus rattus*, 40% for* Peromyscus yucatanicus,* and 42.8% for* Ototylomys phyllotis*) with an average parasite load of 3.15 ± 1.98 eq-parasites/mg.

In this study, no significant association between the studied factors and the infection with* T. cruzi* was found: origin (synanthropic 17.14% vs. wild 9.09%; OR 2.27, 95%CI 0.60 - 8.62); sex (female 16.32% vs. male 3.44%; OR 5.46, 95%CI 0.77 - 38.28), and age (adult 14.58% vs. juvenile 6.66%; OR 2.39, 95%CI 0.47 - 12.01).

## 4. Discussion

Small mammals and especially rodents have been recognized as one of the main reservoirs of several diseases of public health importance in different parts of the world [3]. The results of this study demonstrate the presence and circulation of* T. cruzi* in chronically and actively infected synanthropic and wild rodents of a rural community in southeast Mexico. The presence and circulation of this parasite in the region suggest that transmission to the studied species was through the vector* Triatoma dimidiata*, which is the only vector species of Chagas disease in the Yucatan Peninsula [15]. However, due to the carnivorous, insectivorous, and licking behavior of rodents, oral infection cannot be ruled out [16].

In this study, the synanthropic species with the highest capture frequency were* Rattus rattus* and* Mus musculus* (Table 1). It is documented that those species of rodents and other organisms with synanthropic behavior are common in different regions where agricultural activities are carried out and in the urbanized areas [2, 17]. These same species of rodents have been found in other studies in the Yucatan region [6, 18].

Of the two synanthropic species captured, only in* R. rattus* positive samples were found* T. cruzi* (14.3% positivity in blood samples and 8.7% positivity in heart tissue samples, Table 2), of which only an individual was positive for both types of samples. Other studies carried out in rural communities in Yucatan have reported the presence of infection with* T. cruzi* in* R. rattus* and* Mus musculus* with similar frequency of infection [19].

The presence of synanthropic rodents infected with* T. cruzi* does not imply that the population is going to be irremediably infected; however, these rodents represent a potential risk to public health, since the combination of other factors such as an increase in density of the population of rodents and vectors, the growth of urbanization and agricultural activity, and the presence of more than one reservoir could favor a greater dissemination of the disease in rural communities [2, 20].

This frequency of positivity was similar to those found by Pinto et al. [21] in Ecuador, who reported 11.4% of positivity for* Rattus rattus* and 0% for* Mus musculus*. In contrast, these frequencies differ with those reported by Yefi-Quinteros et al. [22] in Chile, who found a positivity of 83.6% in* Rattus rattus* (5/6). Although Yucatan and the regions of Chile are endemic to Chagas disease [4], this difference in the frequency of infection in synanthropic rodents may be related to the difference in the number of vectors that exist in both regions, since unlike Yucatan, in which there is a vector species of the disease (*T. dimidiata*) [15], in Chile up to four vectors have been reported to be involved in the transmission of* T. cruzi* (*Triatoma infestans*,* Mepraia spinolai*,* Mepraia gajardoi,* and* Mepraia parapatrica*) [23]. Therefore, many vectors could increase the probability of infection in rodents. Other factors that could contribute to this positivity in the samples could be the number of reservoirs, the density of the population of vectors, the density of the susceptible population, or the existence of programs of control of vectors in the regions, and the capacity of vector to adapt to humans habitats [2, 20, 24].

On the other hand, of the 5 wild species of rodents that were captured, only in* Ototylomys phylloty* (25% of blood and 42.85% of heart) and* Peromyscus yucatanicus* (40% of heart) positive samples were found. Although* Heteromys gaumeri* was the rodent with the highest capture frequency, positive samples were not found (Table 2). This greater proportion of positivity in the wild rodents compared to synanthropic rodents could be indicating the presence, maintenance, and circulation of* T. cruzi* in the forest region adjacent to the study site. The presence of* T. cruzi* in forest environments has been reported in different endemic zones for the disease. Studies conducted in forest regions of Argentina reveal a prevalence of wild rodents infected with* T. cruzi* between 6.9% and 18.7% with a major number of species infected (4 species) [25]. In Chile, frequencies of positivity have been reported in blood samples of wild rodents of around 41% to 45% [26] similar to those found in this study. It has been documented that adult triatomines belonging to rural areas can also participate in the transmission of parasite in wild animals, even in the absence of colonization of dwellings [27]. It is important to note that vectors such as* T. dimidiata* or* T. mexicana* are not strictly domiciled and can be found in a wide variety of environments, including domestic, peridomestic, and forest, different from other triatomines such as* T. barberi* that has a greater capacity to establish themselves inside homes [20, 28, 29].

The results of this study evidence the transmission cycle that occurs between the hosts/reservoirs (rodents), the vector (*T. dimidiata*), and the pathogen (*T. cruzi*) at the study site. These interactions are confirmed by the finding of* T. cruzi* amastigote nests in wild and synanthropic rodent species in different sites of Yucatan Peninsula [5, 18]. The presence of* T. cruzi* in the blood of rodents indicates that it is in the acute phase of infection with parasitic loads of 4.8 parasites/mL on average. In heart tissue, parasite loads were 3.15 eq-parasites/mg (chronic infection). In both the acute and chronic phases of the infection, the proportion of positivity was the same for the two types of samples examined (Table 2). These loads coincide with those reported in rodents naturally infected in rural areas of Chile (2.9 to 6.2 parasites / mL) [22, 30]. It has been observed that in the acute phase of the infection, the parasitaemia can fluctuate with time and pass quickly to the chronic phase, maintaining low parasitic loads during this phase [30]. Regardless of the phase of infection in the rodents, they represent a risk for the human population that lives in rural communities because there are favorable conditions for the prosperity of rodents and the availability of food sources for the vector [29].

Although a higher frequency of infected females was observed compared to infected males, no significant association was found between sex and* T. cruzi* infection. However, the importance of females in the maintenance of the agent in the forest and semiurbanized areas should not be underestimated, since the vertical transmission of* T. cruzi* to the offspring has been reported; therefore, the absence of measures that control or limit the growth of rodent populations could increase the level of infection among the population of rodents in the short term [31].

The absence of significant differences between the behavior of rodents (synanthropic or wild) and* T. cruzi* infection seems to indicate that the presence of* T. cruzi* remains stable within the global population of rodents, regardless of their habitat, showing the role of rodents as reservoirs of the disease in the region.

The identification of reservoirs for* T. cruzi* and other diseases transmissible by rodents (i.e.,* Hymenolepis diminuta* and* Leptospira interrogans*) [6] in rural communities is important to understand the dynamic of diseases. With this knowledge, strategies can be implemented for the control of reservoirs host and the vectors, to reduce the dispersion of the agent in the rural areas with greater risk [32].

## 5. Conclusions

The* T. cruzi* parasite circulates actively and chronically in synanthropic and wild rodents captured at the study site. The synanthropic species* Rattus rattus* and the wild species* Ototylomys Phylloty* and* Peromyscus yucatanicus* were the species with the highest frequency of* T. cruzi* infection. Those species represent a potential risk for the transmission of Chagas disease among the inhabitants of the community where these rodents are present. The identification of the main reservoirs of* T. cruzi* in rural areas is important for the implementation of prevention and control strategies of the Chagas disease, in which the control of vectors and reservoirs should be considered.

## Figures and Tables

**Table 1:** Total number of rodents and frequency (%) of sex (female/male) and age (adult/juvenile) of synanthropic and wild rodents captured in a rural community of southwest Mexico.

**Species**	**Total**	**Female** **n (**%**)**	**Male** **n (**%**)**	**Adult** **n (**%**)**	**Juvenile** **n (**%**)**
**Synanthropic**					
*Rattus rattus*	24	10 (42%)	14 (58%)	10 (42%)	14 (58%)
*Mus musculus*	24	17 (71%)	7 (29%)	20 (83%)	4 (17%)
**Wild**					
*Ototylomys phyllotis*	8	5 (63%)	3(37%)	7 (88%)	1 (12%)
*Peromyscus yucatanicus ∗*	8	6 (86%)	1 (14%)	3 (43%)	4 (57%)
*Sigmodon toltecus*	1	1 (100%)	0	1 (100%)	0
*Heteromys gaumeri ∗∗*	17	9 (69%)	4 (31%)	7 (54%)	6 (46%)
*Peromyscus leucopus*	1	1 (100%)	0	0	1 (100%)
***Total***	**83**	**49 (63**%**)**	**29 (37**%**)**	**48 (62**%**)**	**30 (38**%**)**

*∗* 1 and *∗∗*4 samples without sex and age information.

**Table 2:** Frequency (%) of *Trypanosoma cruzi* positive infection diagnosed by qPCR in blood and heart tissue samples of synanthropic and wild rodents captured in a rural community in southwest Mexico.

**Species**	**Blood samples** **positive**	**Parasite/** **µ** **L** **Mean ±SD (range)**	**Heart tissue samples** ** positive**	**eq-parasite/mg** **Mean ±SD (range)**
**Synanthropic**				
*Rattus rattus*	3/21 (14.28%)	4.54 ±1.29 (3.09-5.57)	2/23 (8.69%)	4.70 ±3.68 (2.10-7.31)
*Mus musculus*	0/4 (0%)		0/21 (0)	
**Wild**				
*Ototylomys phyllotis*	1/4 (25%)	5.58	3/7 (42.85%)	2.70 ±0.92 (1.68-3.47)
*Peromyscus yucatanicus*	0/3 (0%)		2/5 (40%)	2.27 ±1.15 (1.45-3.09)
*Sigmodon toltecus*	0/1 (0%)		0/1 (0%)	
*Heteromys gaumeri*	0/8 (0%)		0/10 (0%)	
*Peromyscus leucopus∗*	-		0/1 (0%)	
**Total**	**4/41 (9.75**%**)**	**4.80 ±1.17 (3.09-5.58)**	**7/68 (10.29**%**)**	**3.15 ±1.98 (1.68-7.31)**

*∗* Without blood sample.

## Data Availability

The laboratory results data used to support the findings of this study are available from the corresponding author upon request.
